# Bioinformatics Tools for Plant Genomics

**DOI:** 10.1155/2008/910474

**Published:** 2009-06-11

**Authors:** Gary R. Skuse, Chunguang Du

**Affiliations:** ^1^Bioinformatics Program, Department of Biological Sciences, Rochester Institute of Technology, Rochester, NY 14623, USA; ^2^Science Informatics Program, Department of Biology and Molecular Biology, Montclair State University, Montclair, NJ 07043, USA

The articles in this special issue reflect a convergence of developments in the fields of bioinformatics and plant genomics. Bioinformatics has its roots vaguely seated in the early 1980s, a time when personal computers began appearing in research laboratories and researchers began recognizing that those computers could be used as tools to organize, analyze and visualize their data. In the ensuing years bioinformatics tools began appearing at various sites including the European Molecular Biology Laboratory, the Molecular Biology Research Resource at the Dana-Farber Cancer Institute in the mid 1980s, the National Center for Biotechnology Information (NCBI) in 1988, the Genome Database Project at Johns Hopkins University in early 1989, and in countless laboratories throughout the world. These last efforts resulted in the development of many of the tools described in this special issue.

Progress and interest in plant genomics have been accelerating since the time in late 2000 when the genome of *Arabidopsis thaliana* was published. Since then many genome sequencing projects have been undertaken that include poplar (*Populus*), grape (*Vitis*), the moss *Physcomitrella*, the biflagellate algae *Chlamydomonas* and several globally crucial crop plants such as corn (Maize) and rice (*Oryza*). However, as we have witnessed on numerous occasions, determining the sequence of a genome is only the first step toward understanding genome organization, gene structure, gene expression patterns, disease pathogenesis and a host of other features of both scientific and commercial interests. Computational tools of genomic annotation and comparative genomics must be applied to gain a useful understanding of any genome.

In this special issue we present a collection of papers that together describe a powerful and impactful toolbox of applications and resources for plant genomic analysis. Among those articles you will find a description of research performed by the Mexican headquartered Generation Challenge Programme (GCP) which led to the GCP Platform (Bruskiewich et al.). This research support tool supports a number of data formats and web services and provides access to high performance computing facilities and platform-specific middleware collectively designed to support crop science research.

Probably one of the most promising empirical tools for investigating gene expression developed in the last 15 or so years is that of microarray technology. While the technology has become commonplace, with tools for generating and hybridizing arrays available to all, the analysis of microarray-derived data has been challenging. Many laboratories have struggled not only with this challenge but also with the task of sorting through the plethora of analytical tools available in an effort to find the ones that may be best suited to their own work. In this issue there are two reviews by Page and Coulibaly which examine and describe bioinformatics tools for inferring functional information from plant microarray data. Together these papers step the reader through a collection of tools, and their applications, for analyzing the expression of single and multiple gene expression profiles.

This theme of microarray analysis is continued in the description of the cross chip probe matching tool (CCPMT) by Page et al. Indeed it expands the readers horizons beyond the analysis of individual microarrays with the ability to associate probes across species. And of course, microarray analysis is facilitated by careful experimental design from the start so Robert Tempelman provides a review of statistical methods used to design efficient two-color microarray experiments. Taken together, these microarray papers provide an overview of the design of microarray experiments and the interpretation of the complex results of those experiments that will be informative for new and experienced laboratorians alike.

Several other novel tools are described herein. One, Blast2GO is a suite of tools for the analysis and functional annotation of plant genomes (Conesa and Goetz). It provides an intuitive interface for identifying functional regions within DNA sequences. Another sequence analysis tool described by da Maia et al. is the SSR locator. That tool enables researchers to identify suitable targets for binding PCR primers in order to ensure that those targets are unique within the genome. It also assists with primer design and has a PCR simulator which facilitates comparisons of hypothetical amplification products among different species.

Another challenge facing scientists today is the need to stay abreast of advances in a field that is progressing rapidly as a consequence of newly available technologies. In order to address this challenge there are two review articles that together provide insights into the discovery of relationships among a varied array of plant species. The first article, by Abdurakhmonov and Abdukarimov, describes the application of association mapping to understanding traits in crop species. Their work is directed toward novices within the crop breeding community in order to expose them to potential problems that they may face and solutions they may employ to overcome those problems. The second article describes the tools available for phylogenetic analyses and the increased use of Bayesian methods in those tools (Aris-Brosou and Xia). Constructing phylogenies has traditionally been a challenge to even the most experienced researcher but modern bioinformatics tools are lowering the bar for those interested in detecting adaptive evolution and estimating divergence among species.

The wealth of information available to researchers today can be overwhelming. In order to address this potential, two papers describe information resources which consolidate and organize related information. PPNEMA is a database resource for those interested in plant-parasitic nematode ribosomal genes (Rubino et al.). That resource allows the user to browse, search and generally explore phytoparasite ribosomal DNA. A second database described in these pages is the MaizeGDB (Lawrence et al.). This resource contains information about *Zea mays* which includes genomic sequences as well as functional information and the tools to explore both.

The body of the papers in this special issue represents the leading edge of plant genomics research. Together they provide the reader with descriptions of the tools and resources necessary to understand and promote advances in this important field.

*Gary R. Skuse* *Gary R. Skuse* *Chunguang Du* *Chunguang Du*

